# Iranian physicians' expectations of telemedicine development and implementation infrastructures in teaching hospitals

**DOI:** 10.3934/publichealth.2019.4.514

**Published:** 2019-11-22

**Authors:** Seyedeh Fatemeh Ghafari, Jamileh Mahdizadeh, Ali Valinejadi, Esmaeil Mehraeen, Ali Mohammadpour, Hamid Bouraghi, Mehdi Kahouei

**Affiliations:** 1 School of Medicine, Semnan University of Medical Sciences, Semnan, Iran; 2 Department of English Language, School of Medicine, Semnan University of Medical Sciences, Semnan, Iran; 3 Social Determinants of Health Research Center, Semnan University of Medical Sciences, Semnan, Iran; 4 Department of Health Information Technology, School of Allied Medical Sciences, Semnan University of Medical Sciences, Semnan, Iran; 5 Department of Health Information Technology, Khalkhal University of Medical Sciences, Khalkhal, Iran; 6 Department of Health Information Technology, School of Allied Medical Sciences, Hamadan University of Medical Sciences, Hamadan, Iran

**Keywords:** telemedicine, physicians, hospitals, teaching, Iran

## Abstract

**Introduction:**

In spite of the fact that telemedicine has various advantages; similarly as in some other data systems, it is essential to investigate clients' perspective of technology. Besides, the clients' awareness and satisfaction of the telemedicine are significant issues that ought to be considered before starting a telemedicine program. The present examination in this way looks to assess Iranian doctors' demeanor and recognition toward the infrastructures of telemedicine development and implementation.

**Methods:**

The participants of this examination included doctors working in health care organizations subsidiary to Semnan University of Medical Sciences during 2019 in Iran. A valid and reliable questionnaire was used in order to evaluate the subjects' attitudes.

**Results:**

The mean score of physicians' attitudes towards human factors was 3.43 ± 0.59, towards educational factors was 3.68 ± 0.94 and towards security factors was 3.50 ± 0.52. Regression analysis showed that there were significant relationships between physicians' knowledge and their attitudes towards human (P < 0.001), educational (P < 0.001) and security (P = 0.046) infrastructures.

**Conclusion:**

the findings of this study show that there are several obstacles that can be reduced through teaching, change-management methods and personal patient-to-provider communication. These techniques can improve acceptance and continuous usage of telemedicine among Iranian physicians.

## 1. Introduction

Telemedicine is one of the main zones in which data and correspondence innovations have critical role. This technology offers another strategy for giving healthcare services benefits crosswise over various topographical areas [1], and is utilized for advancing and encouraging the openness of health care services to individuals who don't have access to such services in their private areas [2]–[4].

In spite of the fact that telemedicine has various advantages, similarly as in some other data systems, it is essential to investigate clients' perspective of technology. Besides, the clients' awareness and satisfaction of the telemedicine are significant issues that ought to be considered before starting a telemedicine program [5]. Truth be told, medical services experts' awareness and view of telemedicine are significant components that can impact its future success [6].

A research has demonstrated that the absence of awareness, abilities, and readiness among clients, alongside the elements, are significant boundaries to the utilization of telemedicine, for example the absence of specialized aptitude, introductory expenses, and repayment issues [7]. Then again, appropriate comprehension of telemedicine, particularly by doctors, is a significant necessity for effective usage and sending of the technology [8].

Researches distinguished different examinations that have been finished to demonstrate the comprehension of telemedicine among clinical staff and patients, for instance, an examination led by Hanson et al. demonstrated that doctors and patients would generally wrinkle their knowledge and empower the utilization of telemedicine technology [5].

An investigation by Zayapragassarazan et al. discovered that just 41% of healthcare professionals knew well about the benefits of telemedicine technologies [9]. Alaboudi's study demonstrated that the mentalities of therapeutic staff toward telemedicine and their impression of the potential dangers of telemedicine were significant elements for choosing or deselecting this technology to be utilized in medical services organizations [10].

The objective of this technology is to reinforce the connection between health care providers in remote territories. Doctors have various discernments about the significant data exchanged between the health care institutions. It is consequently imperative to comprehend doctors' perspectives here, as they are fundamental in a fruitful telemedicine. Further research is expected to comprehend the hindrances against providing and using this technology. The present examination in this way looks to assess Iranian doctors' demeanor and recognition toward the infrastructures of telemedicine development and implementation.

## 2. Methods

### 2.1. Setting

The participants of this examination included doctors working in health care organizations subsidiary to Semnan University of Medical Sciences during 2019 in Iran. In order that findings of this study truly represent the physicians' expectations of telemedicine in Iran, inclusion criteria in this study was physicians who like other physicians in Iran, were both full-time in health institutions and used the patient's electronic records.

### 2.2. Recruitment

Based on inclusion criteria and the Cochrane sample size formula with a 5% error level, 183 physicians were included in the study and selected as sample size. It was enough to represent the physicians' attitudes in Iran. Because the sample size was somewhat similar to that of other studies conducted on physicians in Iran. So that the studies that examined physicians' attitudes alone or with the attitudes of other health care professionals such as nurses, pharmacists, and financial and technical officials, 90 to 120 physicians participated [11],[12].

### 2.3. Ethical considerations

Ethics endorsement was made by the Ethics Committee of Semnan University of Medical Sciences (IR.SEMUMS.REC.1397.282). An introductory letter was set up for distribution alongside the survey record that depicted the objectives of examination and disclosed that answering to the questions was considered as the respondent's agreement to participate in the study. It additionally guaranteed the confidentiality of the answers for the participants.

### 2.4. Tools and measures

In this study, Sadeghi et al. questionnaire was used [13]. Sadeghi used the questionnaire for the feasibility of telepathology. By examining the questionnaire, we found that the questionnaire could be used for the feasibility of telemedicine. The questionnaire consisted of 5 sections and 42 questions. The first section consisted of demographic information which was consisted of 4 questions that measured the individual characteristics of the participants; the second part contained 4 questions that measured the level of awareness; the third section included 22 questions that measured the attitudes of the participants the human resources situation; the fourth part included 4 questions that examined the educational situation; and the fifth part included 8 questions that examined the security requirements. The attitude score was estimated for each item on a 5-point Likert-type scale, in which “extremely low” = 1, “low” = 2, “moderate” = 3, “high” = 4 and “extremely high” = 5. Sadeghi et al. used a test-retest method for reliability of the questionnaire. The correlation coefficient was 0.9. In this investigation, the pilot survey was re-conducted on 35 doctors who had been arbitrarily chosen from various health care organizations. The members of the pilot study were barred from the original study. The Cronbach's alpha coefficients of each noted sections were evaluated as 0.881, 0.897, 0.762 and 0.858, separately, while it was 0.944 for the total survey. The final survey was dispersed among the doctors to be later come back to the researcher.

### 2.5. Data analysis

A frequency dissemination table was utilized for describing the categorical factors as attributes, including demographic characteristics. The mean and standard deviation were determined for every item dependent on the attitude scale, which estimated the participants' awareness and their frames of mind toward the human resources circumstances, the instructive circumstances, and the security prerequisites. The cut-off point was set as 3 (score ≤ 3 considered low and score > 3 considered high) in light of the two higher scores of the 5-point Likert scale (“high” and “extremely high”). A low score demonstrated a negative disposition, while a high score showed a positive attitude and general agreement. The one-example t-test was applied to appear if the scores were fundamentally higher or lower than 4. Linear regression analysis was utilized to break down the information and show the connection between the scores of the questionnaire and the inspected attributes. SPSS-16 software was utilized to depict and examine the information at the significance level of 0.05.

## 3. Results

### 3.1. Demographic characteristics

The results indicated that 70.9% of subjects were male and 29.1 were female; mean age was 42.32 ± 5.59 years old; mean work experience was 12.72 ± 6.26 years; 70% of the participants were specialists and 30% were general practitioners.

### 3.2. Awareness and attitude towards telemedicine infrastructure

**Figure 1.**
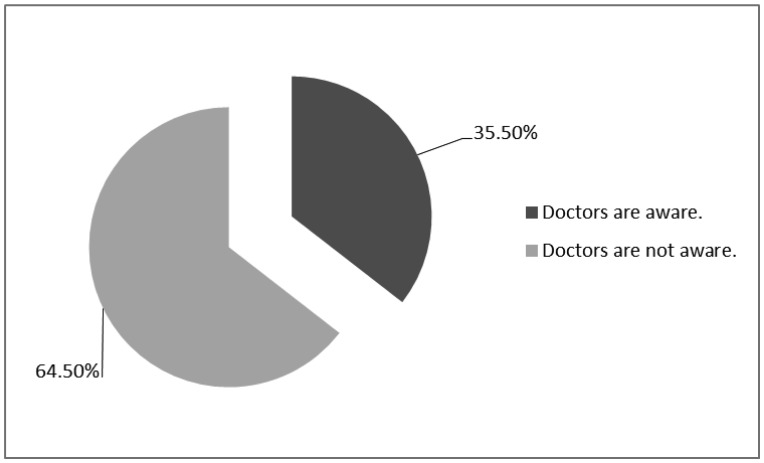
Doctors'awareness of telemedicine.

35.5% of physicians were aware of telemedicine (Figure 1). The mean score of physicians' attitudes towards human factors was 3.43 ± 0.59, towards educational factors was 3.68 ± 0.94 and towards security factors was 3.50 ± 0.52. 81.8% of the physicians had positive attitudes towards human infrastructures, while 18.2% of them had negative attitudes. The results showed that 82.7% of the doctors had positive attitudes towards educational infrastructures and 17.3% of the participants had negative attitudes. The findings indicated that 86.4% of the subjects had positive attitudes towards security infrastructures, while 13.6% of them had negative attitudes. The mean score obtained from physicians' attitudes was 196 ± 30.39.

### 3.3. Inferential statistics

**Figure 2.**
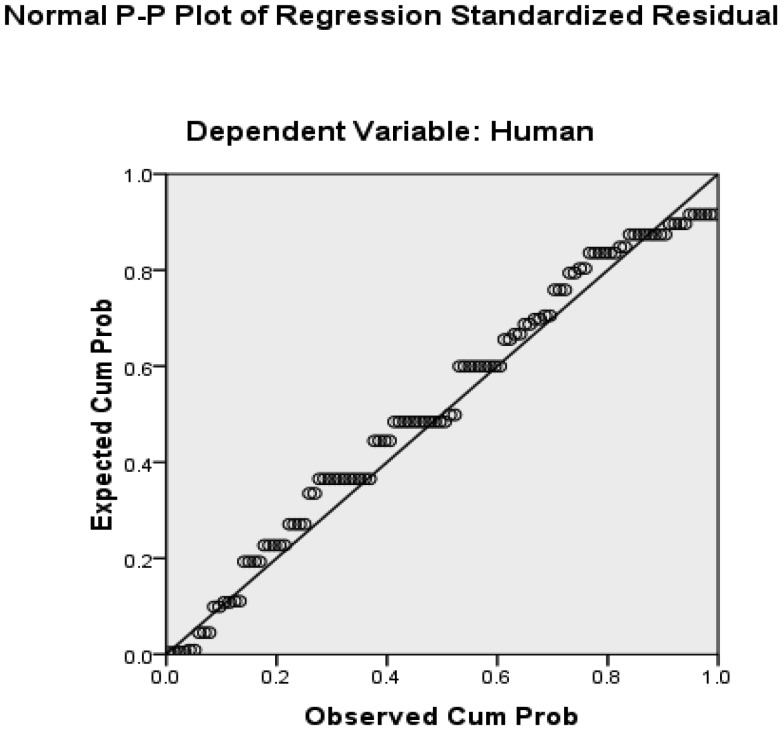
Plot of regression standardized residual between physicians' awareness and their attitudes towards human infrastructures in telemedicine.

The findings showed that there were significant relationships between gender and physicians' awareness (P = 0.038) and their attitudes towards human (P = 0.040) and educational (P = 0.47) components in telemedicine. The results also indicated that there was no significant relationship between other characteristics of physicians and their knowledge and attitudes (P > 0.05) (Table 1). Regression analysis showed that there were significant relationships between physicians' knowledge and their attitudes towards human (P < 0.001, Beta = 0.422), educational (P < 0.001, Beta = 0.334) and security (P = 0.046, Beta = 0.191) infrastructures (Table 2). Plot of regression standardized residual showed that physicians' awareness had the strongest relationship with the attitudes towards human infrastructure in telemedicine (Figure 2).

**Table 1.** P-values of relationships between the participants' characteristics and their awareness and attitudes towards to components of telemedicine.

Characteristics	Awareness	Human	Education	Security
Sex	0.38	0.040	0.047	0.864
Age	0.120	0.167	0.742	0.889
Work experiences	0.112	0.085	0.772	0.703
Specialty	0.788	0.666	0.123	0.417

**Table 2.** Regression analysis between the physicians' awareness and their attitudes towards to components of telemedicine.

components of telemedicine	R	B	Std. Error	t	P-value
Human	0.422	0.253	0.052	4.841	P < 0.001
Education	0.334	0.322	0.087	3.686	P < 0.001
Security	0.191	0.102	0.051	2.017	0.046

## 4. Discussion

In the present investigation, the findings demonstrated that overally few doctors were aware of telemedicine. Correspondingly, in the investigations performed by Torab-Miandoab et al., the findings demonstrated that doctors had little information about telemedicine [14]. In another examination, Rezaei et al. expressed that in spite of the fact that specialists had constrained information and experience of telemedicine, a large number of them were keen on utilizing it [15]. Correspondingly, the examination by Scott Kruse et al. demonstrated that the best hindrance to the execution and acceptance of telemedicine was the absence of clinicians' awareness about telemedicine [2]. In this manner, the results of the present examination are in line with those of different investigations.

The results additionally demonstrated that the participants understood the benefits of telemedicine at a moderate level. In such manner, Ray et al. demonstrated that economical view point has a positive and critical effect on the adoption of telemedicine [16]. Nonetheless, the examination directed by De La Torre-Díezi et al. in a systematic review demonstrated that telemedicine can decrease the expenditures, but not fully [17]. Along these lines, the findings of the present investigation are in line with those of past investigations.

The findings suggested that most of physicians (81.8%) considered the human factors essential to the development and the implementation of telemedicine. It seems that the participants believed that the human framework of telemedicine like the technical foundation was changed and complicated. It usually incorporated an intraorganizational and an interorganizational blend of clinicians, clinical support work force, physicists, programmers and IT experts, administrative support staff and so on. Likewise, those who were legitimately engaged with telemedicine commonly were connected to other work forces associated with financial administration, information systems management, inquiries, and a bunch of patient care activities [18]. The results showed that gender and, more importantly, physicians' awareness of telemedicine had a significant impact (P < 0.001, Beta = 0.422) on their attitudes towards human factors in telemedicine implementation.

All innovations require preparing. Training should be continuous, particularly where the staff's turnover is high [19]. The findings of this study showed that most of the doctors (82.7%) paid special attention to the training of human resources. The results showed that the subjects believed that the staff should be appropriately trained: not only for turning on the devices, but also for utilizing them successfully for consultation, training and management purposes. Findings showed that physicians' attitude toward education in telemedicine has been significantly (P < 0.001, Beta = 0.334) influenced by their knowledge.

Absence of patient trust implied that patients would not disclose precise and complete data, which weakened the quality of care. Low quality of care would decrease the certainty of the both providers and consumers of telemedicine [20]. The findings indicated that most of physicians (86.4%) believed that both patient physical security and patient data safety were essential to build the trust between health care providers and patients and also for using telemedicine. Moreover, the findings showed that this attitude could be influenced (P = 0.046, Beta = 0.191) by the level of physicians' knowledge.

The significance of fulfilling the implementation of safety strategies and procedures in telemedicine was affirmed in several studies [21]–[22]. In this manner, our outcomes were in accordance with the results of previous studies, for example Kamal's study showed that physicians in Pakistan believed that keeping up the privacy of patient data and also the documentation were the most significant elements to set up a protected system for telemedicine [6].

The implications of this research are outside the common obstacles of the implementation of telemedicine. First, the findings showed that Iran should make national eHealth bodies to direct policy and strategy, information security, lawful and moral issues, interoperability, social and language issues, foundation, financing, just as for populace wellbeing following and assessment. Second, Iran should research how telemedicine is effectively and exceptionally being adjusted worldwide to build up a thorough telemedicine model that will be the best conceivable, and adjust this model to the World Health Organization.

This study had two limitations. In the first place, the desires might have been varied from genuine encounters, because the doctors' frame of mind was inspected before implementing telemedicine. Second, in spite of the exertion made by the researchers, limited number of physicians had an interest in taking part in the investigation. The restricted participation might be because of an absence of individual enthusiasm for the subject of investigation or the time limitations in the healthcare centers. Hence, the answers rate was low, and the generalizability of the outcomes to a bigger populace may be influenced. Future research is expected to design technology health care models that consolidate telemedicine to accomplish access to extensive and harmonized patient care.

## 5. Conclusion

Although the usage of telemedicine is common in numerous nations, the findings of this study showed that there were several obstacles that could be reduced through teaching, change-management methods and personal patient-to-provider communication. These techniques can improve acceptance and continuous usage of telemedicine among Iranian physicians.
